# RGF Gene Family Analysis and Functional Evidence for RGF8-Mediated Salt Stress Tolerance in Brassica Species

**DOI:** 10.3390/biology14030281

**Published:** 2025-03-10

**Authors:** Shi-Wei Wang, Fan-Li Zhang, Ru-Yu Jiao, Fan-Xuan Nie, Yi-Lin Zhu, Xi-Jin Huang, Wen-Qing Tan, Qian Yang, Xin-Hong Liu, Li-Li Liu, Duo-Yan Rong, Zhi-Xiang Liu, Qi-Jun Xie

**Affiliations:** 1 Hunan Provincial Key Laboratory of Forestry Biotechnology, College of Life and Enviromental Sciences, Central South University of Forestry and Technology, Changsha 410004, China; shiweiwang2000@163.com; 2 Hunan Research Center of Heterosis Utilization in Rapeseed, Crop Research Institute, Hunan Academy of Agricultural Sciences, Changsha 410125, China; ferry922@163.com (F.-L.Z.); ylzhu0323@163.com (Y.-L.Z.); 16673273280@163.com (X.-J.H.); twq8391290618@163.com (W.-Q.T.); yqhzau@hunaas.cn (Q.Y.); xinhong1220@126.com (X.-H.L.); 3 Longping Branch, College of Biology, Hunan University, Changsha 410125, China; 15386217990@163.com (R.-Y.J.); 22010901018@mail.hnust.edu.cn (F.-X.N.); 4 Hunan Key Laboratory of Economic Crops Genetic Improvement and Integrated Utilization, School of Life and Health Science, Hunan University of Science and Technology, Xiangtan 411201, China; liulili276@163.com; 5 Yuelushan Laboratory, Hongqi Road, Changsha 410125, China; 6 Hunan Provincial Engineering Research Centre of Lily Germplasm Resource Innovation and Deep Processing, College of Life Sciences and Chemistry, Hunan University of Technology, Zhuzhou 412007, China; rongduoyan@163.com

**Keywords:** small peptide, salt stress, RGF8, *Brassica napus* L.

## Abstract

*Brassica napus* L. is a vital oil crop and exhibits notable salt tolerance, making the enhancement of this trait crucial for cultivation in saline soils. Uncovering its underlying mechanism may further heighten the salt tolerance of *B. napus*. In this study, we conducted a genome-wide analysis of the *Root Meristem Growth Factor* (RGF) gene family in *Brassica* species and identified *BnaRGF8* as a key regulator of salt tolerance. Under 200 mM NaCl treatment, all RGF genes were induced, with *BnaRGF8* showing the greatest upregulation. This response was consistently observed across 10 genotypes, indicating a universal trend in *B. napus*. Functional characterization revealed that chemically synthesized *BnaRGF8* alleviated salt-induced growth inhibition by restoring primary root elongation and lateral root formation. Additionally, *BnaRGF8* preserved membrane integrity and maintained cell division under salt stress, establishing itself as a novel anti-salt peptide in *B. napus*.

## Simple Summary

This study investigates the *Root Meristem Growth Factor* (RGF) gene family in cruciferous plants, with a particular focus on BnaRGF8 and its role in enhancing salt tolerance in *Brassica napus*. The objective is to improve the cultivation of rapeseed in saline–alkali soils, thereby increasing crop yields. The results demonstrate a significant upregulation of BnaRGF8 expression in response to 200 mM NaCl treatment across 10 genotypes. Furthermore, the chemically synthesized BnaRGF8 peptide effectively alleviates salt-induced growth inhibition, promotes root elongation and lateral root formation, preserves cell membrane integrity, and stimulates cell division. These findings suggest that BnaRGF8 could serve as a promising novel salt-tolerant peptide. Notably, BnaRGF8 represents the first RGF family member associated with salt tolerance in rapeseed, providing valuable insights for enhancing crop salt tolerance through molecular breeding and addressing the growing global challenge of soil salinization.

## 1. Introduction

Soil salinization has become a major threat to global agricultural productivity, significantly limiting crop growth and yields. Enhancing salt tolerance in crops is essential for sustainable agriculture, particularly in saline-affected regions. As an important oil crop, *Brassica napus* L. exhibits notable salt tolerance, making it a promising candidate for cultivation in saline soils. Understanding the molecular mechanisms underlying salt tolerance in *B. napus* is crucial for developing effective strategies to enhance this trait.

To date, metabolic processes represented by several genotypes associated with salt tolerance have been identified across various crops, including salt transport (such as SOS, HKT, NHX) [1], reactive oxygen species scavenging (like SOD, APX) [2], transcription regulation (such as DREB/CBF) [3,4], and osmotic adjustment (like P5CS) [5,6,7]. For instance, SOS1 encodes a plasma membrane sodium/proton exchanger that extrudes Na^+^ from the cell, reducing sodium toxicity [8,9]. Similarly, SOD and APX alleviate oxidative damage from salt stress by scavenging reactive oxygen species [2,10]. While P5CS synthesizes proline to maintain cellular osmotic pressure [11], further boosting plants’ salt tolerance. It is well known that salinity, through various pathways such as cell cycle regulation, inhibits the growth of meristematic cells. However, understanding the salt response is still limited, especially in rapeseed crops, such as *B. napus*.

Secreted peptides, which serve as important internal signals, play key roles in both growth regulation and stress [12]. For example, RALF22/RALF23 serves as a sensing element for cell wall integrity, relaying salt stress signals into the cell [13]. In parallel, RALF22 inhibits root growth in absence of salt [14]. CLAVATA3(CLV)/EMBRYO-SURROUNDINGREGION-RELATED (CLE), known for its role in regulating meristem differentiation [15], is also proved to modulate salt tolerance by elevating ABA synthesis in the face of salt stress [12]. Notably, increasing numbers of small peptides have been reported to have the dual role of regulating tolerance and growth.

To date, 11 *Root Meristem Growth Factors* (RGFs) have been identified in Arabidopsis thaliana, where they play essential roles in various aspects of plant development, including the maintenance of the root meristem, root hair development, lateral root formation, and gravitropism in both roots and hypocotyls [16,17,18]. RGFs are part of a family of plant-specific peptide signals critical for sustaining root meristem activity and regulating cell division. Like other secreted signaling peptides, mature RGF polypeptides are generated through proteolytic cleavage from precursor proteins. These precursor proteins typically consist of 79–182 amino acids, including an N-terminal signal peptide that directs the protein to the secretory pathway, and a C-terminal conserved RGF domain [16,17,18]. The mature peptides, composed of 13–16 amino acid residues, often undergo post-translational modifications such as tyrosine sulfation and proline hydroxylation.

In addition to their roles in development, RGFs have been implicated in plant stress responses, including tolerance to salt stress [19]. However, the exact functions of RGFs in salt tolerance remain largely unexplored, particularly in economically important crops such as *B. napus*. While the RGF gene family has been extensively studied in Arabidopsis, its distribution, evolutionary dynamics, and functional significance in *B. napus* are not well understood. Furthermore, the specific contributions of individual RGF genes to salt stress tolerance in *B. napus* remain to be determined.

In this study, we performed a comprehensive genome-wide analysis of the RGF gene family in *B. napus* to identify its members, evolutionary relationships, and expression patterns. We then focused on *BnaRGF8*, demonstrating its critical role in enhancing salt tolerance through functional characterization. These findings provide novel insights into the molecular mechanisms underlying salt tolerance in *B. napus* and offer potential targets for improving crop resilience to saline environments.

## 2. Materials and Methods

### 2.1. Plant Material and Growth Conditions

A total of 10 different varieties of *B. napus* [20] and the widely cultivated accession ZS11 were selected for this study. Seeds exhibiting full grains and uniform texture were sterilized by immersion in 75% ethanol for 1 min, followed by three rinses with distilled water. Sterilized seeds were placed in seed germination boxes (8 × 8 × 8 cm) lined with four layers of filter paper (50 seeds per box). Healthy seedlings were transferred to germination boxes containing Hoagland liquid medium supplemented with NaCl (200 mM), RGF8 peptide (1 μM), or NaCl + RGF8, and grown under controlled conditions (22 °C, 16 h light/8 h dark cycle) for 4 days. Roots from treated seedlings were sampled, flash-frozen in liquid nitrogen, and stored at −80 °C for subsequent RT-qPCR analysis.

### 2.2. Identification and Evolutionary Analysis of the Brassica Species RGF Gene Family

RGF protein sequences from *A. thaliana* were retrieved from TAIR (https://www.arabidopsis.org)(accessed on 10 December 2024). Homologous sequences in *B. napus* (BnIR database: https://yanglab.hzau.edu.cn/BnIR/download_ZS11_v0) (accessed on 10 December 2024), *B. oleracea* (http://brassicadb.cn/#/Download/Indexof/download_genome/Brassica_Genome_data/Braol_JZS_V2.0) (accessed on 10 December 2024), and *B. rapa* (http://brassicadb.cn/#/Download/Indexof/download_genome/Brassica_Genome_data/Brara_Chiifu_V3.5) (accessed on 10 December 2024) were identified using BLASTP (E-value < 1 × 10^−10^). Candidate RGF proteins were validated against the InterPro HMM model PF21529 (https://www.ebi.ac.uk/interpro/) (accessed on 12 December 2024). ProtParam (https://web.expasy.org/protparam/) (accessed on 12 December 2024) was used to analyze physicochemical properties. Multiple sequence alignment of RGFs from Brassica and *A. thaliana* was performed with MUSCLE (https://www.ebi.ac.uk/Tools/msa/muscle/) (accessed on 18 December 2024). A maximum-likelihood phylogenetic tree was constructed using IQ-tree and visualized with Evolview (https://www.evolgenius.info/evolview-v2/) (accessed on 18 December 2024). Subcellular localization was predicted using WoLFPSORT (https://wolfpsort.hgc.jp/) (accessed on 19 December 2024) and Cell-PLoc 2.0 (http://www.csbio.sjtu.edu.cn/bioinf/plant/) (accessed on 19 December 2024).

### 2.3. Chromosomal Localization and Collinearity Analysis of the Brassica Species RGF Gene Family

RGF gene locations were mapped to chromosomes using TBtools-II (V2.154) and genomic coordinates from Brassica GFF3 annotation files. Collinearity relationships within *B. napus* and between Brassica species were identified using MCscanX (V1.8) with default parameters.

### 2.4. Conserved Motifs, Domains, and Gene Structure Analysis of the Brassica Species RGF Gene Family

Conserved motifs in RGF proteins were identified using MEME Suite (https://meme-suite.org/meme/) (accessed on 20 December 2024). Gene structures (intron-exon organization) and cis-acting elements were visualized with TBtools and PlantCARE (https://bioinformatics.psb.ugent.be/webtools/plantcare/html/) (accessed on 20 December 2024), respectively.

### 2.5. Analysis of Expression Patterns of BnRGFs Based on Transcriptome Data

Tissue-specific, stress-responsive, and hormone-induced expression data for *BnRGFs* were obtained from the BnIR database (https://yanglab.hzau.edu.cn/) (accessed on 21 December 2024). Heatmaps were generated using TBtools to visualize expression patterns.

### 2.6. Analysis of Expression Patterns of BnRGFs Under Salt Stress

Gene-specific primers (Supplementary Table S4) were designed with Primer Premier 5. Total RNA was extracted using the FlaPure Plant Total RNA Extraction Kit (Genesand Biotech, Beijing, China), and cDNA was synthesized with All-In-One 5X RT MasterMix (Applied Biological Materials, Richmond, Canada). qRT-PCR was performed on a Bio-Rad CFX96 system using *BnaACT7* as the reference gene. Relative expression levels were calculated using the 2^−ΔΔCq^ method.

### 2.7. BnaRGF8 Peptides Treatment

BnaRGF8 (DYSKFRRRRPVHN) was synthesized by DGpeptide company (Hangzhou, China). All peptides were dissolved in distilled water to a concentration of 10 mM and were aliquoted to avoid repeated freeze–thaw cycles, and were stored at −20 °C.

Four-day-old ZS11 seedlings were treated with 200 mM NaCl, 1 μM BnaRGF8, or NaCl + RGF8 for 4 days. Root phenotypes (primary root length, lateral root length, and lateral root number) were quantified using an Epson scanner (Epson, Tokyo, Japan) and WinRHIZO software ((https://regent.qc.ca/assets/winrhizo_software.html (accessed on 20 December 2024), Regent Instruments Inc., Quebec, Canada). The experimental design consisted of a combination of four treatments: WT (ZS11), WT + NaCl (ZS11 treated with 200 mM NaCl), BnaRGF8 (ZS11 treated with 1 μM BnaRGF8), and BnaRGF8 + NaCl (ZS11 treated with 1 μM BnaRGF8 + 200 mM NaCl).

### 2.8. PI Staining

Root tips (5 cm) were stained with 1 mg/mL propidium iodide (PI) for 15 min, washed three times with ddH2O, and imaged on a Zeiss LSM880 confocal microscope (Zeiss, Oberkochen, Oberkochen, Germany) (excitation: 553 nm). Membrane damage rates were calculated as the ratio of PI-positive cells to total cells in the root apex region.

### 2.9. Ionic Leakage Rate

Seedlings (0.1 g) were immersed in ultrapure water or 200 mM NaCl for 1 h, rinsed, and soaked in 10 mL water for 2 h. Conductivity (E1) was measured with a DDS-11A conductivity meter. Samples were boiled (95 °C, 15 min), cooled, and conductivity (E2) was remeasured. Relative electrical conductivity (%) was calculated as (E1/E2) × 100.

### 2.10. Analysis of Expression Patterns of BnSOSs Under BnRGF8 Treatment

Four-day-old ZS11 seedlings were treated with 1 μM BnaRGF8 for 4 days. Roots from treated seedlings were sampled, flash-frozen in liquid nitrogen, and stored at −80 °C for subsequent RT-qPCR analysis. The experimental design consisted of a combination of two treatments: WT (ZS11), BnaRGF8 (ZS11 treated with 1 μM BnaRGF8). Roots from treated seedlings were sampled, flash-frozen in liquid nitrogen, and stored at −80 °C for subsequent RT-qPCR analysis. Gene-specific primers (Supplementary Table S4) were designed with Primer Premier 5. Total RNA was extracted using the FlaPure Plant Total RNA Extraction Kit (Genesand Biotech), and cDNA was synthesized with All-In-One 5X RT MasterMix (Applied Biological Materials). qRT-PCR was performed on a Bio-Rad CFX96 system using *BnaACT7* as the reference gene. Relative expression levels were calculated using the 2^−ΔΔCq^ method.

### 2.11. Statistical Analysis

All statistical analysis was performed using the One-way ANOVA test with a significant difference via GraphPad Prism 9.0 (* *p* < 0.05; ** *p* < 0.01) developed by GraphPad Software company (GraphPad Software, La Jolla, CA, USA).

## 3. Results

### 3.1. Identification and Evolutionary Analysis of Brassica Species RGF Gene Family

In this study, we performed a comprehensive analysis of the RGF gene family in three Brassica species, including *Brassica napus* (Bn), *Brassica rapa* (Br), and *Brassica oleracea* (Bo). A total of 47 RGF genes were identified across the three species (Table 1), with 21 members from *B. napus*, 14 from *B. rapa*, and 12 from *B. oleracea*. The gene sequences ranged in size from 78 to 274 amino acids, with protein molecular weights varying from 8.9 to 31.1 kDa. The protein length distribution showed that most RGFs in these species were between 80 and 130 amino acids in length, consistent with the characteristic size of the small peptide family. The average molecular weight (MW) for BnRGFs was 14.6 kDa, while BnRGFs and BoRGFs had average MWs of 13.1 and 14.6 kDa, respectively. The isoelectric points (pI) of the identified RGF proteins ranged from 7.1 to 10.9, with most proteins showing a slightly basic pI, suggesting that these proteins may function in alkaline environments or have structural features that stabilize them in such conditions.

Protein localization within the cell is intricately linked to its function; therefore, predicting the cellular localization of a protein is indispensable for investigating gene function. Subcellular localization prediction using WoLFPSORT indicated that RGF proteins were primarily localized in several key cellular compartments: vacuole (vacu), extracellular space (extr), chloroplast (chlo), cytoplasm (cyto), nucleus (nucl), and mitochondria (mito). The majority of RGFs in all three species were predicted to localize to the vacuole (10 members in *B. napus*, 6 in *B. rapa*, and 5 in *B. oleracea*). This localization suggests that the RGFs might be involved in processes such as cell wall remodeling, ion homeostasis, or signaling within vacuolar compartments. Noteworthy, the predicted subcellular localization refers to the precursor form of the peptide, which requires cleavage to generate the mature form. The vacuolar localization of the precursor highlights its potential role in growth regulation or stress tolerance, two processes closely associated with vacuolar dynamics. Additionally, a number of RGF genes were predicted to be extracellular (6 in *B. napus*, 5 in *B. rapa*, and 4 in *B. oleracea*), further indicating potential roles in intercellular communication or stress responses. Other RGFs were predicted to localize to chloroplasts, which is consistent with their potential involvement in photosynthesis-related processes, while several RGFs were predicted to reside in the cytoplasm or nucleus, suggesting roles in signaling pathways and gene regulation.

**Figure 1:**
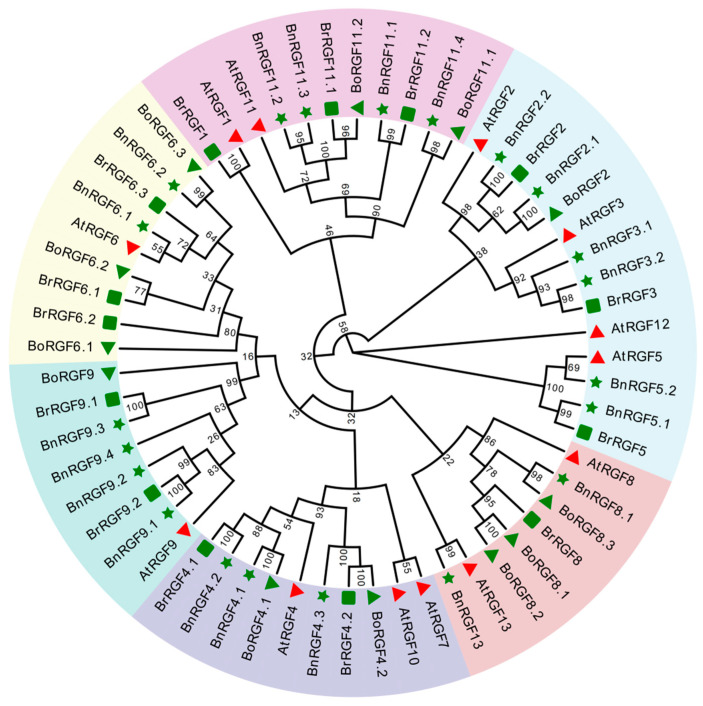
Phylogenetic tree of the RGF gene family in *Brassica napus*, *Brassica rapa*, and *Brassica oleracea*. The tree was constructed using the maximum likelihood method, with Arabidopsis (*Arabidopsis thaliana*) RGF homologs as references. Bootstrap values are indicated at key nodes to assess the reliability of clustering. Genes are labeled based on their species origin: Bn (*Brassica napus*), Br (*Brassica rapa*), Bo (*Brassica oleracea*), and At (*Arabidopsis thaliana*). In this figure, the light purple background of the leaves represents *RGF1s*/*RGF11s*, the light blue background represents *RGF2s/RGF3s/RGF5s/RGF12*, the light red background represents *RGF8s/RGF13*, the light yellow background represents rgf6s, the dark purple background represents *RGF4s/RGF7/RGF10*, and the dark blue background represents *RGF9s*. The leaf labels are distinguished by different shapes representing the respective plant species’ *RGFs*: red triangles denote Atrgfs, green triangles denote *BoRGFs*, stars denote *BnRGFs*, and rectangles denote *BrRGFs*. Moreover, Bootstrap values falling within the range of 0 to 50 are not rendered for display.

Evolutionary analysis is highly beneficial for studying gene functions, inter-species evolutionary relationships, as well as genetic diversity and variations. The phylogenetic analysis of the RGF gene family in *B. napus*, *B. rapa*, and *B. oleracea* revealed distinct evolutionary relationships among members of this family. A maximum likelihood (ML) phylogenetic tree was constructed, integrating homologous sequences from *A. thaliana* as references to infer evolutionary divergence and gene expansion events (Figure 1).

The RGF family in the three Brassica species exhibited conserved clustering patterns with their *A. thaliana* counterparts. Specifically, the tree highlights subclades corresponding to *ATRGF* homologs, such as *AtRGF4*, *AtRGF5*, *AtRGF8*, and others. Gene duplications and diversification within the Brassica genus were evident, as shown by multiple paralogs present in each species. For example: *BnRGF4* (including *BnRGF4.1*, *BnRGF4.2*, and *BnRGF4.3*) clustered closely with *BoRGF4* and *BrRGF4*, suggesting species-specific expansions after the divergence of Brassica species from their common ancestor. *BnRGF5.1* and *BnRGF5.2* showed close evolutionary relationships with *BrRGF5* and *AtRGF5*, implying functional conservation. Interestingly, certain clades, such as those containing *BnRGF8.1*, *BoRGF8.1*, and *BrRGF8*, displayed stronger sequence similarity to *AtRGF8*, which might indicate conserved roles in growth regulation. In contrast, other subclades, such as those with *BnRGF13* and *AtRGF13*, were more species-specific, reflecting possible neofunctionalization.

### 3.2. Chromosomal Localization and Collinearity Analysis of the Brassica Species RGF Gene Family

Chromosomal localization of the RGF gene family in *B. napus* was analyzed to explore the genomic distribution of these genes. A total of 21 RGF genes were mapped across the chromosomes of *B. napus*, covering both the A and C subgenomes. These genes were unevenly distributed, with clusters observed in specific chromosomal regions. The chromosomal localization indicates that 21 *BnRGFs* are distributed across 14 out of the 19 chromosomes in the *B. napus* genome, including 11 in A subgenome and 10 in C subgenome. In the A subgenome, RGF genes were predominantly located on chromosomes A01, A02, A03, A04, A06, and A09. Similarly, in the C subgenome, RGF genes were distributed on chromosomes C02, C03, C04, C05, C08, and C09. The distribution patterns suggest a possible role of gene duplication and chromosomal rearrangements in the evolution of the RGF gene family in *B. napus*. Furthermore, the localization of RGF genes in clusters implies potential functional or regulatory conservation within the family. These results provide a foundation for further functional analysis of RGF genes in *B. napus* (Supplementary Figure S1A).

The chromosomal distribution of RGF genes in *B. rapa* and *B. oleracea* was analyzed to elucidate their genomic organization and potential evolutionary patterns. A total of 28 RGF genes were identified and mapped onto the chromosomes of both species, with distinct patterns of localization observed. In *B. rapa*, 14 *BrRGFs* are located on 7 chromosomes out of 10, and in *B. oleracea*, 12 *BoRGFs* are positioned on 8 chromosomes out of 9.

In *B. rapa*, RGF genes were primarily located on chromosomes A01, A02, A03, A04, A06, A08, and A09. Representative examples include *BrRGF6.3* on chromosome A01 and *BrRGF11.1* on chromosome A09. The clustering of genes on certain chromosomes, such as A02 and A06, suggests the possibility of gene duplication events contributing to the expansion of the RGF gene family in this species.

In *B. oleracea*, RGF genes were found on chromosomes C01, C02, C03, C04, C05, C07, C08, and C09. Notable examples include *BoRGF6.3* on chromosome C01 and *BoRGF11.1* on chromosome C08. Similar to *B. rapa*, clustering patterns were also observed, particularly on chromosomes C02 and C05, indicating evolutionary conservation or divergence in genomic organization between the two species (Supplementary Figure S1B).

Overall, the conserved chromosomal localization of the gene family across the A and C subgenomes of the allopolyploid and related species suggests strong evolutionary constraints, likely driven by functional importance and selective pressure to maintain regulatory or structural integrity.

### 3.3. Collinearity Analysis of the Brassica Species RGF Gene Family

Collinearity analysis serves as a pivotal tool for comprehensively exploring genome architecture and evolution, facilitating the elucidation of genetic relationships and evolutionary trajectories among diverse biological species. Examination of collinearity within *B. napus* unveiled 16 RGF syntenic gene pairs. Furthermore (Supplementary Figure S2A), inter-genomic collinearity analysis involving *A. thaliana*, *B. rapa*, and *B. oleracea* revealed 39 RGF syntenic gene pairs (Supplementary Figure S2B), while 41 RGF syntenic gene pairs were identified in the collinearity analysis among *B. napus*, *B. rapa*, and *B. oleracea* (Supplementary Figure S2C).

In plant genomes, tandem repeats and segmental duplications have been instrumental in expanding gene family members and facilitating the emergence of novel functions during evolutionary processes. To elucidate the evolutionary scenarios within the RGF gene families of *B. napus*, *B. rapa*, and *B. oleracea*, we investigated tandem repeats and segmental duplication events. Surprisingly, no tandem repeat genes were observed in *B. napus*, *B. rapa*, or *B. oleracea*. Among the 47 Brassica species RGF genes studied, most events have been found to originate from whole genome duplications or segmental duplication events. These findings strongly indicate the pivotal role of segmental duplication in the evolutionary trajectory of RGF genes.

To investigate the evolutionary relationships and genomic organization of the RGF gene family in *B. napus*, a genome-wide collinearity analysis was performed. The analysis identified significant collinear relationships among RGF genes distributed across the A and C subgenomes of *B. napus*. A total of 21 RGF genes were located on 19 chromosomes, with A subgenome chromosomes labeled as A01–A10 and C subgenome chromosomes labeled as C01–C09. Notable collinear relationships were observed between A07 and C06, as well as A01 and C01, suggesting duplication events that contributed to the expansion of the RGF gene family. The density and position of RGF genes on individual chromosomes were also visualized. Regions with higher gene density were observed on chromosomes such as A02 and C05, while other chromosomes, such as A05 and C09, displayed relatively sparse gene distribution. This pattern reflects the evolutionary history of polyploidization and chromosomal rearrangements in *B. napus*. The results provide valuable insights into the genomic organization and evolutionary dynamics of the RGF gene family in *B. napus*, highlighting the role of gene duplication and chromosomal rearrangement in shaping the current distribution of this gene family(Supplementary Figure S2).

The localization and collinearity analysis collectively highlight the prevalence of segmental duplications in the gene family suggesting that whole-genome duplication events and chromosomal rearrangements have driven its expansion, providing functional redundancy and opportunities for adaptation through neofunctionalization and subfunctionalization.

### 3.4. Conserved Motifs Analysis of the Brassica Species RGF Gene Family

**Figure 2:**
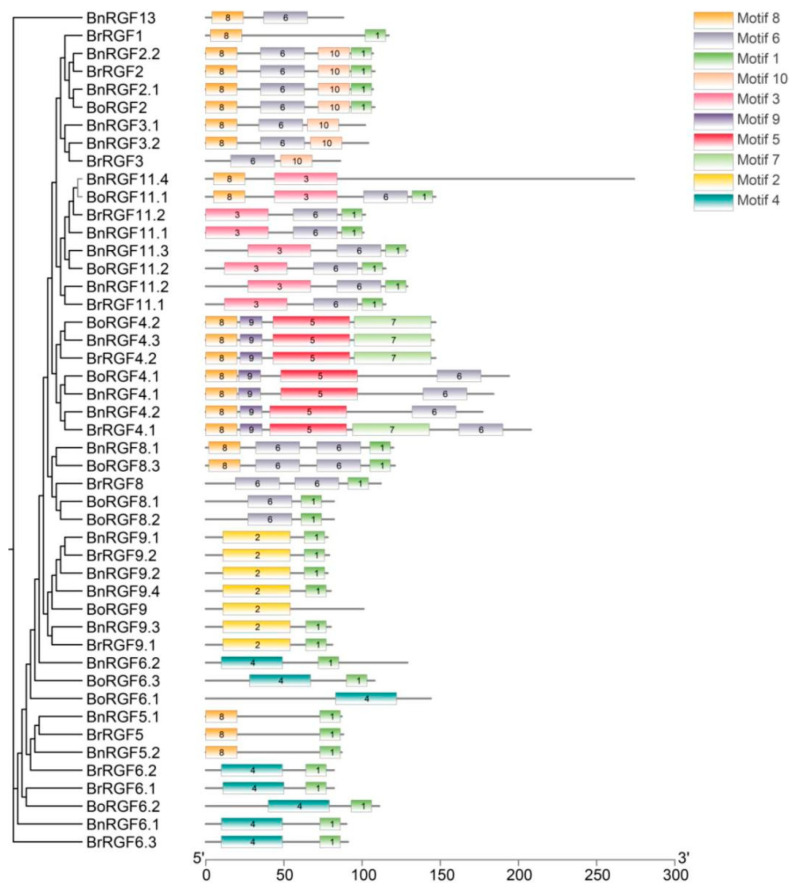
Phylogenetic relationships and conserved motif distribution of RGF genes in *Brassica napus, Brassica rapa*, and *Brassica oleracea*. The phylogenetic tree (**left**) shows the clustering of RGF genes, while the conserved motif compositions (**right**) are depicted as colored boxes. Motifs identified by MEME are numbered 1–10 and represented by distinct colors as indicated in the legend. The x-axis indicates the protein length (in amino acids). The conserved motifs suggest core functional domains, while subgroup-specific motifs imply functional diversification among RGF gene family members.

In order to predict protein function and discover the relationship between protein structure and function, conserved motif analysis is performed. The phylogenetic relationships revealed clustering of RGF genes into distinct subgroups, reflecting evolutionary divergence among the three species. Motif analysis showed that members within the same subgroup shared similar motif patterns, suggesting functional conservation (Supplementary Table S1). Motif 1, Motif 2, and Motif 3 were present in most RGF genes, indicating a high level of conservation for these motifs within the RGF gene family of Brassica species. In contrast, some motifs, such as Motif 8 and Motif 10, were found to be subgroup-specific, which may represent functional specialization or adaptation to specific physiological processes in the respective species (Figure 2).

### 3.5. Cis-Acting Element Analysis of the Brassica Species RGF Gene Family Promoter Regions

To better understand the regulatory mechanisms of RGF genes in *B. napus*, *B. rapa*, and *B. oleracea*, cis-regulatory elements (CREs) in the 2 kb upstream promoter regions were analyzed. This analysis revealed an extensive distribution of diverse CREs, reflecting their involvement in various stress responses, hormonal signaling, and developmental processes. Stress-responsive elements, such as ARE (anaerobic induction), LTR (low-temperature responsiveness), and MBS (drought-inducibility), were identified across multiple RGF genes, suggesting their potential roles in abiotic stress adaptation. Additionally, hormone-related CREs, including ABRE (abscisic acid responsiveness), TGA-element (auxin responsiveness), and CGTCA-motif (MeJA responsiveness), were widely distributed, indicating that RGF genes are regulated by plant hormones in response to environmental cues. Light-responsive elements, such as Box 4, G-box, and AE-box, were observed in nearly all analyzed promoter regions, highlighting the potential roles of RGF genes in photoperception and light-mediated physiological responses. Furthermore, development-related CREs, such as CAT-box (meristem expression) and O2-site (zein metabolism regulation), were also detected, suggesting involvement in tissue-specific and growth-related gene regulation (Supplementary Figure S3).

### 3.6. Analysis of Expression Patterns of BnRGFs Based on Transcriptome Data

**Figure 3:**
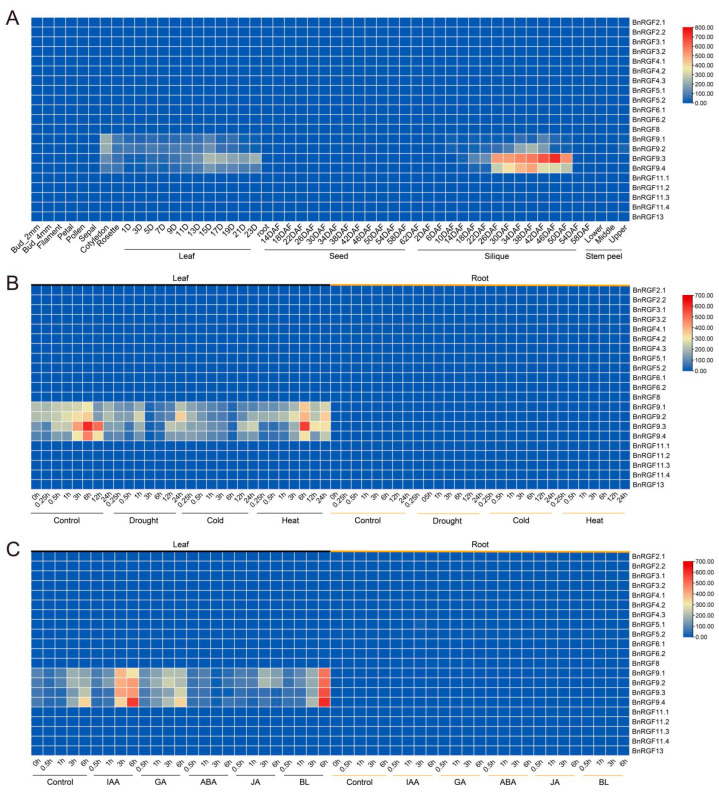
Analysis of Expression Patterns in *BnRGFs*. (**A**) Tissue-specific expression patterns of *BnRGFs*. (**B**) Expression patterns of *BnRGFs* under stress conditions. (**C**) Expression patterns of *BnRGFs* under hormone treatments. In Figure 3A, the notations ‘N + D’ and ‘N + DAF’ represent the N day and the N day after flowering, respectively. Within Figure 3C, the abbreviations correspond to specific phytohormones: IAA for indole-3-acetic acid, GA for gibberellic acid, ABA for abscisic acid, JA for jasmonic acid, and BL for brassinolide. The expression of *BnNRAMPs* was normalized and represented in TPM (transcripts per kilobase of exon model per million mapped reads), and log_2_(TPM + 1) was used to construct the heatmap diagram.

Expression patterns represent a pivotal aspect elucidating gene function. To explore the RGF genes’ roles further, we constructed a heatmap displaying RGF gene expression patterns utilizing publicly available data from the BnIR website (https://yanglab.hzau.edu.cn/)(accessed on 21 December 2024). The tissue-specific expression patterns of the RGF gene family in *B. napus* were analyzed across various tissues and developmental stages using heatmap visualization. The expression profiles revealed distinct spatial and temporal expression patterns, indicating potential functional specialization among the RGF gene members. Several RGF genes, including *BnRGF6.1* and *BnRGF3.2*, showed high expression levels in specific tissues such as petals and sepals, suggesting their roles in floral development. Other members, such as *BnRGF4.2* and *BnRGF9.1*, exhibited elevated expression in early seed developmental stages, as seen in samples from 20 to 40 days after flowering (DAF), implying their involvement in seed formation and maturation processes. In contrast, genes like BnRGF9.3 displayed tissue-specific expression peaks in roots, highlighting their possible roles in root growth and development. Interestingly, some RGF genes, such as *BnRGF5.1* and *BnRGF11.3*, were lowly expressed or nearly undetectable in most tissues, suggesting a possible role in response to specific environmental or developmental cues rather than constitutive expression. These diverse expression profiles underline the functional diversity within the RGF gene family and their contributions to tissue-specific and developmental stage-specific processes in *B. napus* (Figure 3A).

The expression profiles of BnRGF genes in *B. napus* were assessed under various abiotic stress conditions, including cold, heat, and drought, across multiple time points (0.5 h, 1 h, 3 h, 6 h, and 24 h) in leaf and root tissues. The heatmap highlights a diverse range of responses, indicating that BnRGF genes exhibit stress-specific and tissue-specific expression patterns. Notably, *BnRGF4.2* and *BnRGF11.1* displayed significant upregulation in leaf tissues under drought stress at 1 h and 6 h, respectively, suggesting their potential role in drought response mechanisms. Similarly, *BnRGF9.3* and *BnRGF6.1* were strongly induced by cold stress in roots, indicating their involvement in cold tolerance. In contrast, *BnRGF13* was prominently upregulated under heat stress in leaves, suggesting its potential role in heat adaptation. Temporal analysis revealed that certain genes, such as *BnRGF3.2* and *BnRGF9.4*, exhibited rapid and transient induction, while others, like *BnRGF11.4* and *BnRGF5.2*, showed sustained expression over prolonged stress exposure. These results indicate the complexity and specificity of *BnRGF* genes regulation in response to abiotic stresses, emphasizing their potential importance in stress adaptation and resilience in *B. napus* (Figure 3B).

The expression patterns of BnRGF genes under various hormone treatments were analyzed to investigate their regulatory roles in response to hormonal signals. Heatmap visualization demonstrated distinct expression profiles across different treatments, tissues, and time points. In leaf tissues, BnRGF genes showed significant induction under auxin (IAA) and gibberellin (GA) treatments, particularly for *BnRGF9.3* and *BnRGF6.1*, which displayed peak expression levels at 3 h and 6 h, respectively. Jasmonic acid (JA) and brassinosteroid (BL) treatments also elicited notable expression changes in specific genes, such as *BnRGF5.2* and *BnRGF4.3*, indicating their potential involvement in signaling pathways associated with these hormones. In root tissues, several BnRGF genes, including *BnRGF11.4* and *BnRGF13*, were highly responsive to abscisic acid (ABA) and auxin (IAA), suggesting their roles in root development and stress adaptation. The dynamic expression patterns revealed both temporal and tissue-specific regulation, underscoring the functional diversity of BnRGF genes in hormonal signaling networks. These findings provide important insights into the roles of BnRGF genes under hormonal stimuli and establish a foundation for further functional studies on their regulatory mechanisms in hormone-mediated developmental and stress responses (Figure 3C).

### 3.7. Analysis of Expression Patterns of BnRGFs Under Salt Stress

We previously discussed the potential involvement of BnRGFs in abiotic stress responses. Specifically, expression profiling and cis-element analysis suggested their roles in various abiotic stresses. Subcellular localization and collinearity analysis further indicated functional redundancy and potential subfunctionalization within the gene family. In this context, RGFs expressed under normal conditions are likely to be associated with developmental processes, while those with low expression levels may play specialized roles in responding to environmental adversities.

**Figure 4:**
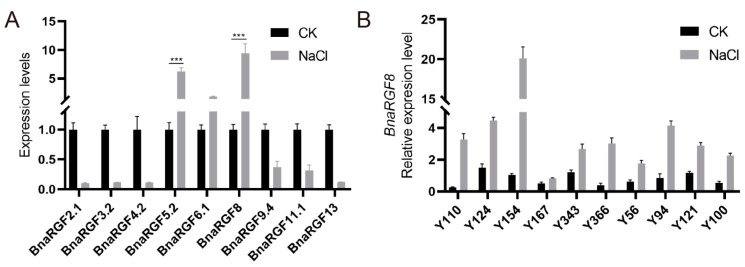
RT-qPCR analysis of BnaRGFs. (**A**) Differential Responses of *BnaRGFs*. Expression Under Salt Stress in Brassica napus seedlings. The expression levels of each *BnaRGF* gene in the NaCl treatment group were normalized to those in the control group (CK). (**B**) *BnaRGF8* Expression Under Salt Stress in Brassica napus seedlings. Each bar data represents mean ± standard error (*n* = 3). Statistical analysis was performed with Student’s *t*-test (*** *p* < 0.001).

A previous study demonstrated that vacuoles and the cell wall are functionally linked to salt stress responses. Interestingly, RGFs are localized to both the apoplast and vacuoles, suggesting their potential involvement in these mechanisms. To explore the response of these genes to salt stress, we measured their expression levels under 200 mM NaCl treatment in ZS11, a widely used control cultivar in *B. napus*. Interestingly, most RGFs are responsive to salt stress, within which two closely related *BnaRGF5* and *BnaRGF8* are upregulated under NaCl treatment (Figure 4A). However, other *BnaRGF* genes showed an opposite trend, implying functional diversity within the family. We focused on *BnaRGF8*, which showed the most pronounced upregulation in exposure to 200 mM NaCl. We tested its expression in 10 additional *B. napus* genotypes and found consistent positive responses across all (Figure 4B). Accordingly, we hypothesize that *BnaRGF8* may broadly be involved in the salt tolerance response of *B. napus*. We tested its expression in another 10 rapeseed accessions with phenotypic variation (Supplementary Table S3) and found that all accessions showed a consistent positive response (Figure 4B). Therefore, we hypothesized that BnaRGF8 may be widely involved in the salt tolerance response of rapeseed.

### 3.8. BnaRGF8 Exhibits a Unique Growth Regulatory Effect

**Figure 5:**
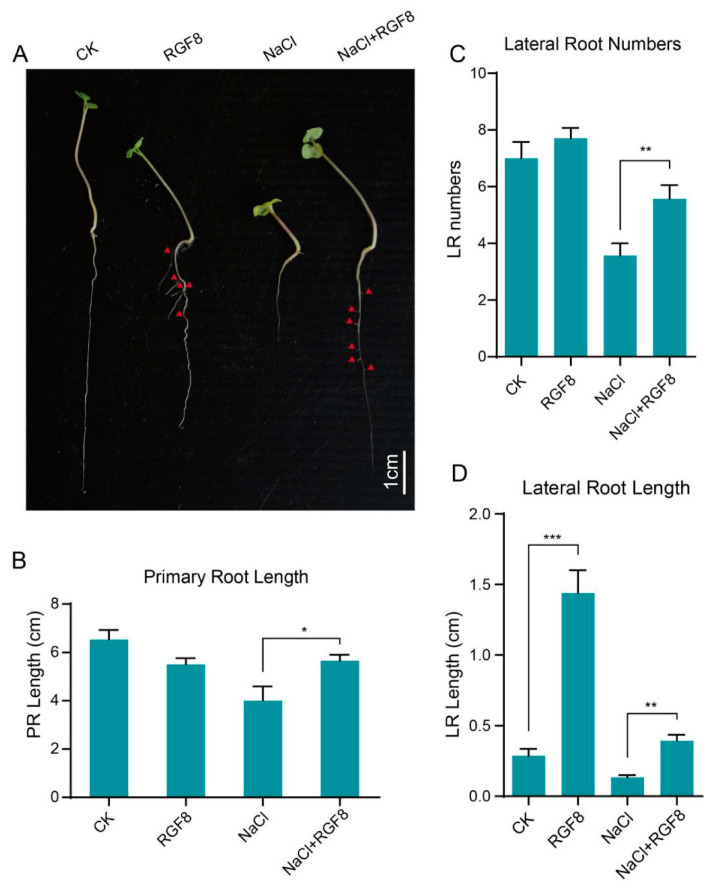
BnaRGF8 alleviates the inhibition of root growth by NaCl. (**A**) ZS11 hydroponic seedlings under different treatment conditions. (**B**) Statistical histograms of the length of the primary root under different treatment conditions. (**C**) The number of lateral roots of ZS11 under different treatment conditions. (**D**) The length of lateral roots of ZS11 under different treatment conditions. Each data point or bar is the mean ± SD of at least 15 independent biological replicates. Statistical analysis was performed with Student’s *t*-test (* *p* < 0.05; ** *p* < 0.01; *** *p* < 0.001).

To verify the above hypothesis, we analyzed the salt tolerance capabilities of BnaRGF8, which showed the greatest response to NaCl treatment. While applied with 1 μM to ZS11 seedlings, BnaRGF8 significantly promoted lateral root elongation (Figure 5A). Moreover, the number of lateral roots was increased after BnaRGF8 treatment, indicating the promoted lateral root formation and elongation. This result conflicts with previously reported functions of *AtRGFs* [21], which include an inhibitory effect of lateral root formation. One possible explanation might be the inherent differences between species, where BnaRGF8 gained distinct functions. Surprisingly, primary root growth was inhibited by BnaRGF8 (Figure 5). Overall, the BnaRGF8 exhibited a phenotype partially opposite to that of its homologous in Arabidopsis, i.e., promoting lateral root growth and inhibiting primary root growth.

### 3.9. BnaRGF8 Significantly Enhances Salt Tolerance in ZS11

Meanwhile, when treated with 200 mM NaCl, BnaRGF8 shows a significant growth-promoting effect both in the primary root and lateral roots. (Figure 5). Specifically, while 200 mM NaCl reduced primary root elongation by nearly 50%, BnaRGF8 almost completely reversed this inhibition, when comparing with BnaRGF8 treatment (Figure 5). Although BnaRGF8 shows a certain promoting effect on lateral root elongation and formation under salt treatment, this promoting effect also exists when BnaRGF8 is applied alone. Therefore, BnaRGF8 may not necessarily alleviate the inhibition of lateral roots caused by salt stress. These results suggest that besides a growth regulator, BnaRGF8 is likely to be a salt-tolerance peptide which specifically counteracts growth inhibition especially in primary root under salt stress.

### 3.10. BnaRGF8 Helps Maintain Cell Membrane Integrity and Cell Division

**Figure 6:**
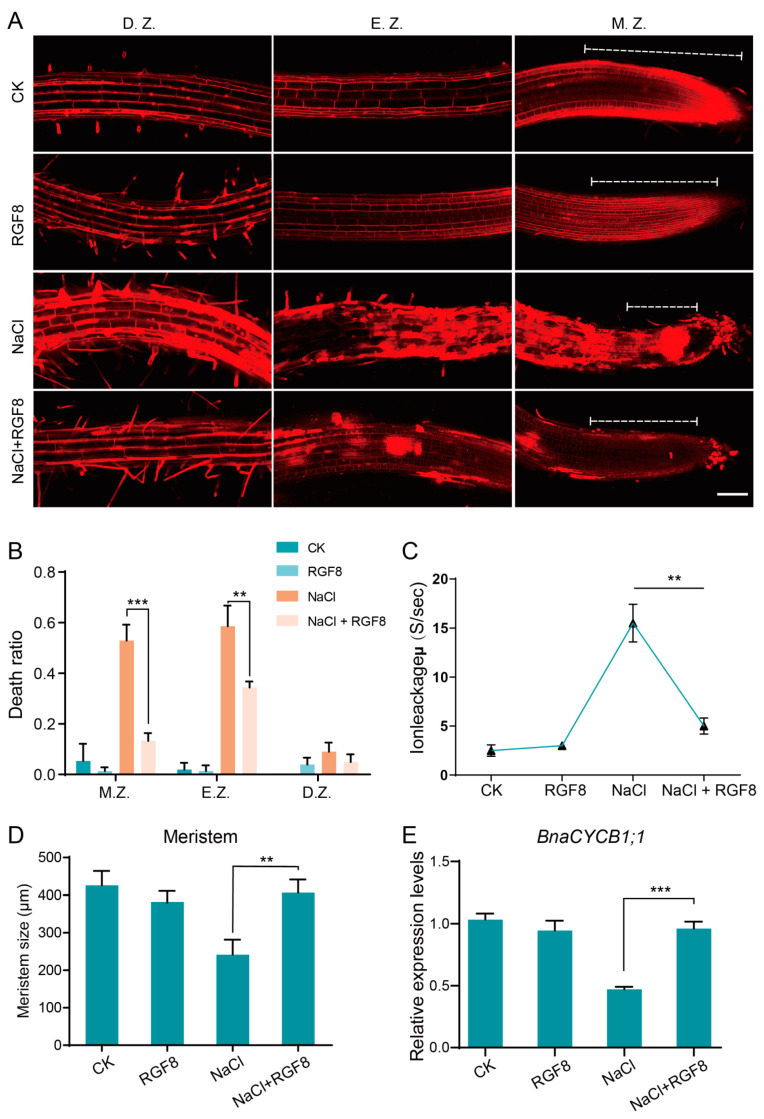
*BnaRGF8* alleviates NaCl-induced root cell breakage rate. (**A**) PI staining of root tips of ZS11 hydroponically grown seedlings under different treatment conditions. D.Z.: maturation zone; E.Z.: elongation zone; M.Z.: meristematic zone. The white dashed line represents the meristematic tissue. Scal Bar = 100 μm. (**B**) Histogram of cell mortality statistics in different zones of root tips of ZS11 hydroponically grown seedlings under different treatment conditions. (**C**) Ion leakage from root cells under different treatment conditions. (**D**) Length of the meristematic zone of the primary root of ZS11 under different treatment conditions. (**E**) Expression of the mitosis marker gene *CYCB1;1* in ZS11 under different treatment conditions. Bar data or bar are the mean ± SD of with independent biological replicates (For (**B**) and (**D**), *n* > 15; Panel (**C**) and (**E**), *n* = 3). Statistical analysis was performed with Student’s *t*-test (** *p* < 0.01; *** *p* < 0.001).

High salt causes damage by disrupting cell membrane integrity, which can be assessed using propidium iodide (PI) dye. When the cell membrane is damaged, PI permeates the membrane, resulting in patchy staining. Otherwise, PI clearly outlines intact cells by using this method. We found that NaCl treatment indeed caused damage to the cell membrane integrity, as patchy PI signals (corresponding to damaged cells) were observed almost throughout the entire root region, particularly in the meristem zone. The integrity of the plasma membrane was also confirmed by assessing ion leakage rates. Leakage of intracellular content caused by damage on the membrane can increase conductivity of the surrounding solution. Monitoring the conductivity dynamics reveals that BnaRGF8 effectively reduced salt-induced ion leakage. This damage may possibly cause impairment in meristematic activity, and lead a reduction in the cell division rate. Indeed, NaCl treatment significantly reduced the meristem size, as well as reducing the expression level of the mitotic marker gene *CYCB1;1*. However, BnaRGF8 notably reversed this trend. Specifically, the patchy signal of PI staining was greatly reduced in meristem, the size of meristem increased to comparable length of untreated one, and *CYCB1;1* expression was restored to similar level of CK (Figure 6). It is worth-noting that BnaRGF8 treatment alone neither showed a clear effect on the meristem size, nor did it enhance *CYCB1;1* expression. Clearly, in *B. napus*, BnaRGF8 was assigned to maintain cells proliferation specifically under salt stress condition, highlighting its salt-tolerance feature rather than growth-promoting effect. Overall, BnaRGF8 helps maintain cell membrane integrity, preserving meristematic cell activity.

## 4. Discussion

Growth and stress tolerance in plants are often seen as opposing forces. Salt stress notably inhibits growth by suppressing cell division [21,22,23], as evidenced by the reduced expression of genes like *CYCA2;1* and *CYCB1;1* under NaCl stress. However, few studies explore how suppressed cell division recovers [21,24]. Our findings on BnaRGF8, a small peptide, indicate it enhances salt tolerance while restoring cell division, challenging the notion of strict antagonism between growth and tolerance. Previous reports have noted that some genes, like *OsDREB1C* and *OsNRT2.3b* in rice, promote both tolerance and growth [25,26]. Although the specific role of *BnaRGF8* remains unclear, particularly without genetic evidence from mutant lines, our results strongly suggest its importance in salt tolerance.

While gene family members are often thought to have similar roles, functional understanding can lag. The *WOX* family, initially recognized for development [27], was later found to regulate stress responses [28,29,30]. Similarly, though RGFs were seen mainly as growth regulators, our research highlights BnaRGF8’s specific role in salt stress tolerance. Under non-stress conditions, BnaRGF8 promotes lateral root formation while inhibiting primary root growth, but its low expression limits its impact on root growth. Salt stress significantly upregulates *BnaRGF8*, suggesting its primary role in growth recovery under stress. Additionally, AtRGF1’s role in regulating ROS homeostasis in Arabidopsis may partially explain this family’s salt tolerance [31].

The sodium ion efflux pathway (SOS pathway) plays a crucial role in plant responses to salt stress and is regarded as one of the key regulatory mechanisms [32,33,34]. This study investigates the impact of BnaRGF8 on the expression level of ZS11, yielding promising and noteworthy results. We observed that treatment with BnaRGF8 significantly upregulated the expression of *SOS3*, while the expression levels of *SOS1* and *SOS2* did not exhibit significant changes (Supplementary Figure S4). These findings suggest that RGF8 may regulate *SOS3* expression via specific signaling pathways, providing novel insights into the potential role of BnaRGF8 in plant salt stress responses. By enhancing *SOS3* expression, BnaRGF8 may facilitate the efflux of sodium ions, effectively maintaining intracellular ion balance and improving plant salt tolerance. These results align with previous studies, offering a fresh perspective on the role of BnaRGF8 in regulating *SOS3* expression and contributing to a deeper understanding of the molecular mechanisms underlying plant salt tolerance. In light of the growing global challenge of soil salinization, this study provides valuable insights for breeding salt-tolerant crops and highlights the potential for modulating RGF8-related signaling pathways to improve crop performance in saline–alkaline environments.

Currently, biotechnological strategies for enhancing salt tolerance are limited, with transgenic methods facing policy restrictions [35]. Secreted small peptides like BnaRGF8 offer a promising alternative, as they can be applied exogenously and mass-produced through fermentation, paving the way for developing biological agents to regulate salt tolerance.

## 5. Conclusions

This study provides a comprehensive analysis of the RGF gene family in *Brassica napus*, revealing its evolutionary conservation and expansion primarily through segmental duplications. Evidence of functional divergence among RGF genes suggests subfunctionalization, enabling specialized roles in various biological processes, including stress responses.

In addition, we identified BnRGF8 as a key regulator of salt tolerance in *B. napus*, marking it as the first anti-salt peptide discovered in this species. While RGF8 specifically promotes lateral root growth rather than primary root growth, its role in enhancing salt tolerance appears to be primarily through preventing membrane damage, which indirectly supports cell division. This hypothesis is supported by ion leakage assays and PI staining. Interestingly, the function of BnaRGF8 in *B. napus* contrasts with that of its homolog in *Arabidopsis thaliana*, highlighting the evolution of distinct functions in different species.

These findings deepen our understanding of the RGF gene family’s evolutionary and functional dynamics and provide valuable insights for improving salt tolerance in crops through molecular breeding.

## Figures and Tables

**Table 1:** Physicochemical indices and subcellular localization predictions of *B. napus* RGF gene family members.

No.	Gene ID	Gene Name	Chromosome	Protein Length (aa)	MW (kDa)	PI	Subcellular Localization Predicted
1	BnaC05T0107900ZS	*BnRGF2.1*	C05	107	12,088.79	7.72	extr
2	BnaA06T0087900ZS	*BnRGF2.2*	A06	107	12,259.97	7.72	extr
3	BnaC02T0445400ZS	*BnRGF3.1*	C02	102	11,637.42	9.64	vacu
4	BnaC02T0480800ZS	*BnRGF4.1*	C02	184	20,836.98	9.89	vacu
5	BnaA02T0330000ZS	*BnRGF3.2*	A02	104	11,837.66	9.59	vacu
6	BnaA02T0357400ZS	*BnRGF4.2*	A02	177	19,888.93	10.13	extr
7	BnaA06T0369300ZS	*BnRGF4.3*	A06	146	16,718.36	10.28	extr
8	BnaA03T0139900ZS	*BnRGF5.1*	A03	87	9859.39	10.58	chlo
9	BnaC03T0162200ZS	*BnRGF5.2*	C03	87	9864.41	10.93	chlo
10	BnaA01T0186400ZS	*BnRGF6.1*	A01	90	9841.59	9.7	chlo
11	BnaC01T0237000ZS	*BnRGF6.2*	C01	129	14,269.5	8.67	chlo
12	BnaC09T0264800ZS	*BnRGF8*	C09	120	13,452.67	9.59	vacu
13	BnaA02T0410000ZS	*BnRGF9.1*	A02	78	8967.3	9.06	vacu
14	BnaC02T0544800ZS	*BnRGF9.2*	C02	78	8888.23	9.63	vacu
15	BnaA06T0297700ZS	*BnRGF9.3*	A06	80	9040.46	10	chlo
16	BnaC03T0528500ZS	*BnRGF9.4*	C03	80	8895.26	10.12	chlo
17	BnaA09T0551200ZS	*BnRGF11.1*	A09	129	14,742.51	7.1	cyto
18	BnaC08T0397700ZS	*BnRGF11.2*	C08	129	14,726.64	9.08	extr
19	BnaA04T0014500ZS	*BnRGF11.3*	A04	101	11,372.69	9.7	nucl
20	BnaC04T0274900ZS	*BnRGF11.4*	C04	274	31,070.06	10.05	chlo
21	BnaA10T0207300ZS	*BnRGF13*	A10	88	10,127.93	9.45	chlo
22	BraA02g042690.3C	*BrRGF1*	A02	116	12,888.96	10.29	chlo
23	BraA06g009850.3C	*BrRGF2*	A06	107	12,259.97	7.72	extr
24	BraA02g036380.3C	*BrRGF3*	A02	85	9706.93	9.63	cyto
25	BraA02g039170.3C	*BrRGF4.1*	A02	207	23,364.93	10.23	extr
26	BraA06g036170.3C	*BrRGF4.2*	A06	146	16,654.32	10.25	extr
27	BraA03g015470.3C	*BrRGF5*	A03	87	9859.39	10.58	chlo
28	BraA08g011940.3C	*BrRGF6.1*	A08	81	9135.73	9.73	chlo
29	BraA03g046980.3C	*BrRGF6.2*	A03	81	8937.58	10.64	chlo
30	BraA01g020060.3C	*BrRGF6.3*	A01	90	9946.69	10.04	chlo
31	BraA02g036300.3C	*BrRGF8*	A02	111	12,702.69	9.91	chlo
32	BraA06g028220.3C	*BrRGF9.1*	A06	80	9040.46	10	chlo
33	BraA02g044770.3C	*BrRGF9.2*	A02	78	8967.3	9.4	vacu
34	BraA09g050400.3C	*BrRGF11.1*	A09	114	13,047.7	9.41	cyto
35	BraA04g001620.3C	*BrRGF11.2*	A04	101	11,363.68	9.7	nucl
36	BolC05g011060.2J	*BoRGF2*	C05	107	12,088.79	7.72	extr
37	BolC02g055000.2J	*BoRGF4.1*	C02	193	22,173.42	9.63	cyto
38	BolC07g036970.2J	*BoRGF4.2*	C07	146	16,723.36	10.28	extr
39	BolC01g024840.2J	*BoRGF6.1*	C01	143	16,065.26	9.3	chlo
40	BolC08g015580.2J	*BoRGF6.2*	C08	110	12,426.39	9.78	chlo
41	BolC01g024810.2J	*BoRGF6.3*	C01	107	12,013.11	10.46	mito
42	BolC02g051410.2J	*BoRGF8.1*	C02	81	9313.53	9.77	nucl
43	BolC02g051420.2J	*BoRGF8.2*	C02	81	9313.53	9.77	nucl:
44	BolC09g028690.2J	*BoRGF8.3*	C09	120	13,448.68	9.59	Vacu
45	BolC03g055880.2J	*BoRGF9*	C03	100	11,199.78	7.98	chlo
46	BolC08g043940.2J	*BoRGF11.1*	C08	114	13,049.68	9.38	cyto
47	BolC04g032180.2J	*BoRGF11.2*	C04	146	16,715.13	9.39	extr

Note: extr: Cell wall; Vacu: Vacuole; chlo: Chloroplast: cyto: Cytosol; nucl: Nucleus; mito: Mitochondrion.

## Data Availability

All data generated or analyzed during this study are included in this published article and its Supplementary Materials.

## Supplementary Materials

The following supporting information can be downloaded at: https://www.mdpi.com/article/10.3390/biology14030281/s1, Figure S1–S4: Chromosomal localization of the Brassica species RGF gene family; Co-linearity relationships of *RGFs* among *B. napus* and three ancestral plants; Cis-regulatory element analysis of the selected 2000 bp upstream promoter regions of Brassica species RGF genes; RT-qPCR analysis of BnaSOSs. Table S1–S4: Conserved motifs of BnRGF protein; Cis-acting elements; *B. napus* used in this study; Primers used in this study.
