# Supplementary material for: RGF Gene Family Analysis and Functional Evidence for RGF8-Mediated Salt Stress Tolerance in Brassica Species
Source: Biology (Basel). 2025 Mar 10;14(3):281. doi: 10.3390/biology14030281 (PMC11939866; doi:10.3390/biology14030281)
Supplement: Supplementary file 1 [file biology-14-00281-s001.zip › biology-3471141-supplementary.pdf]

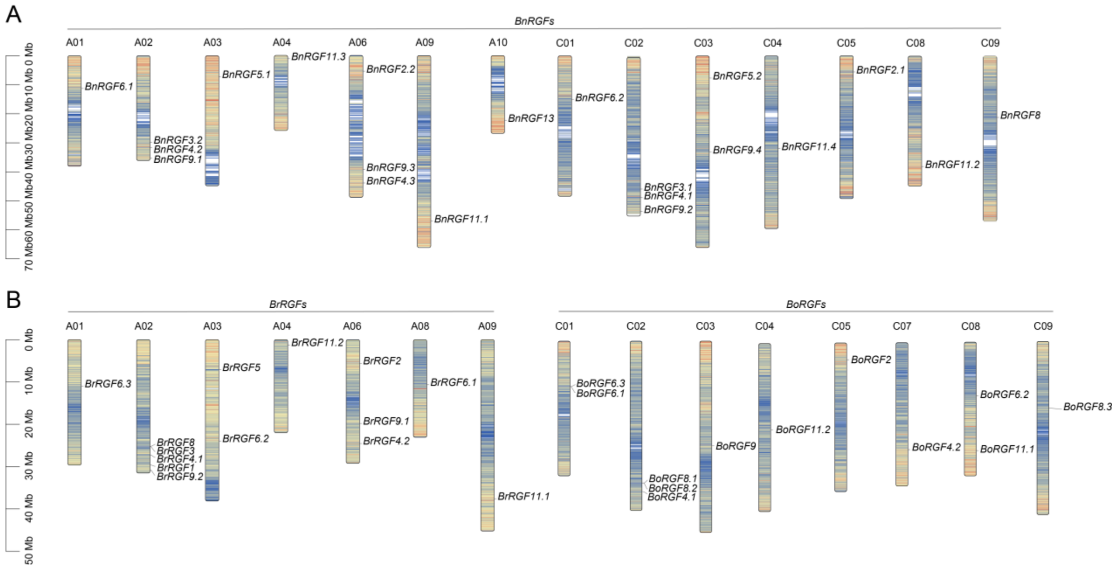

Figure S1. Chromosomal localization of the Brassica species RGF gene family. **(A)** Chromosomal localization in *B. napus*. **(B)** Chromosomal localization in *B. rapa* and *B. oleracea*.

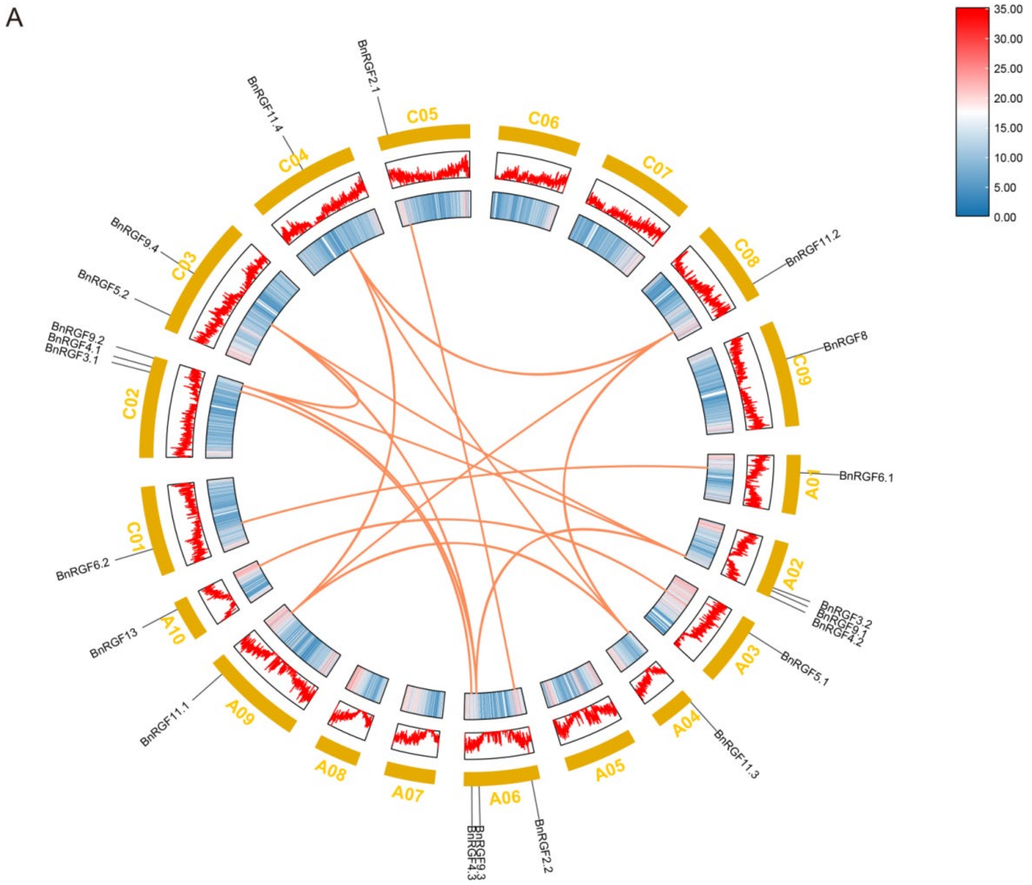

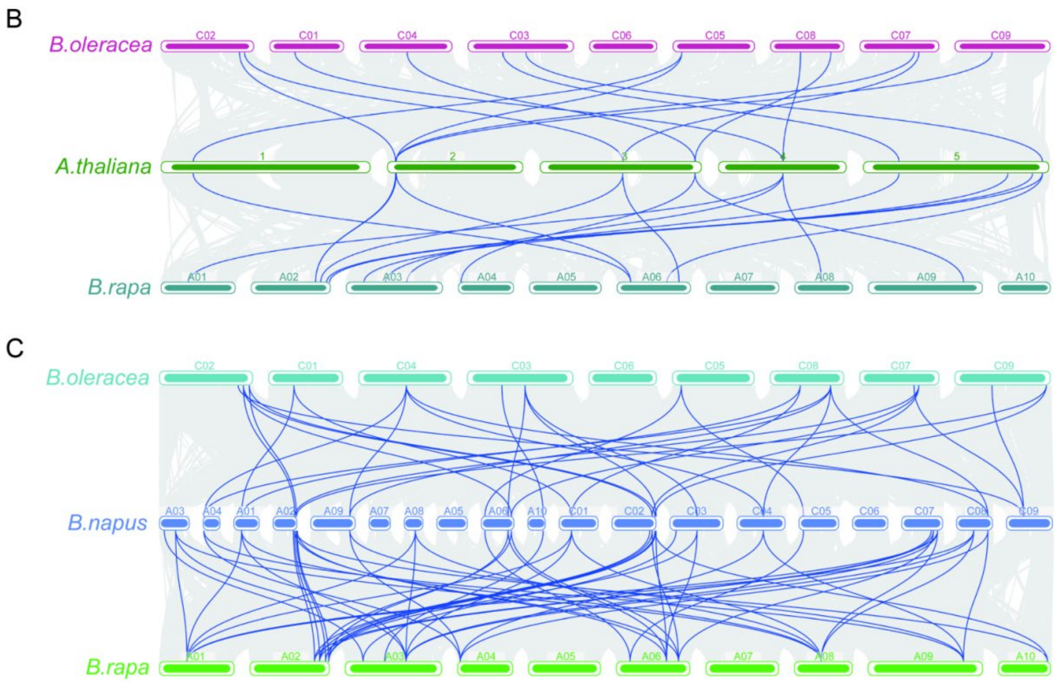

Figure S2. Co-linearity relationships of *RGFs* among *B. napus* and three ancestral plants. **(A)** Co-linearity relationships within the *B. napus* genome. **(B)** Co-linearity relationships among *A. thaliana*, *B. rapa*, and *B. napus* genomes. **(C)** Co-linearity relationships among *B. rapa*, *B. napus*, and *B. oleracea* genomes. Gray lines in the background indicate co-linear blocks among Brassica species, while blue lines represent co-linear RGF gene pairs.

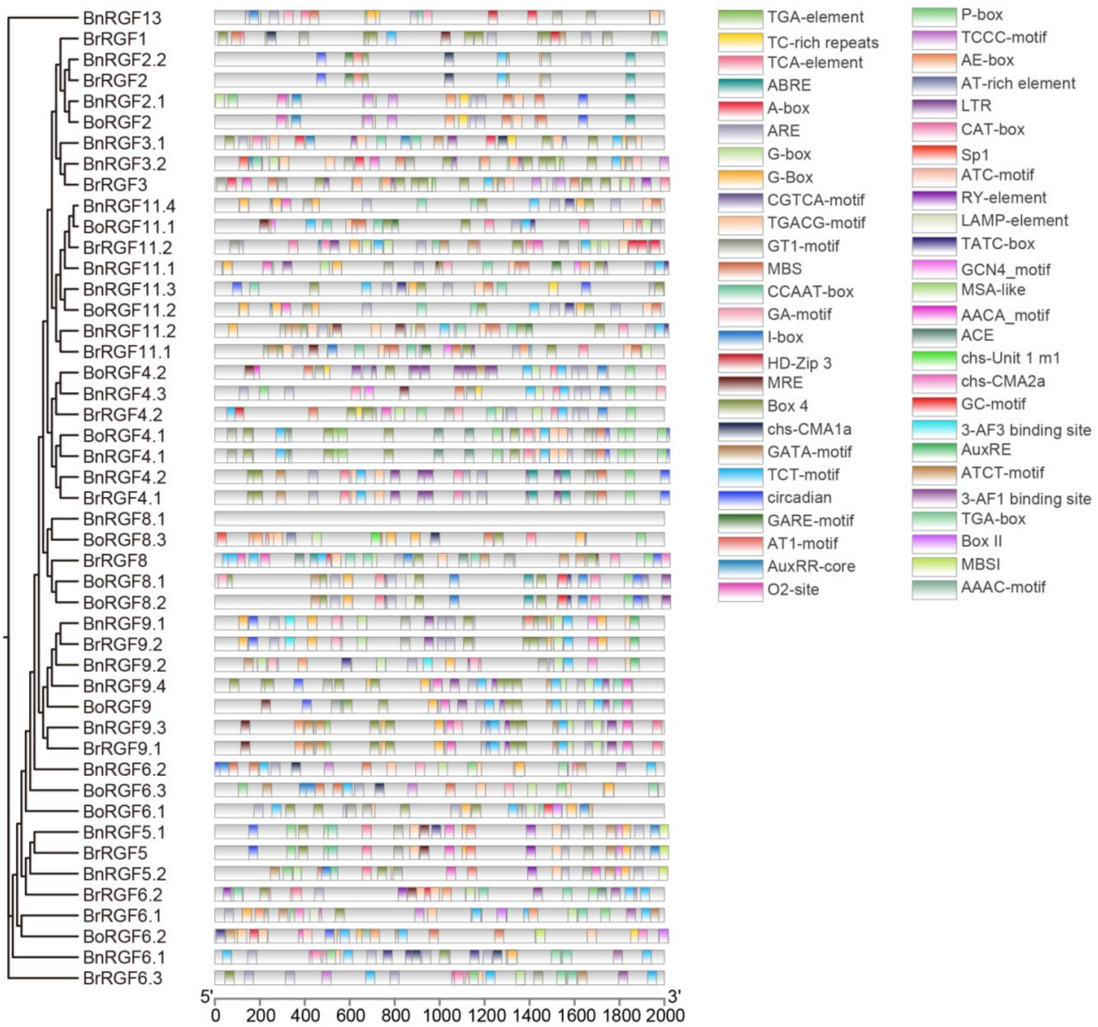

Figure S3. Cis-regulatory element analysis of the selected 2000 bp upstream promoter regions of Brassica species RGF genes. TGA-element:auxin-responsive element; AT-rich element:binding site of AT-rich DNA binding protein (ATBP-1); MSA-like:cis-acting element involved in cell cycle regulation; TC-rich repeats:cis-acting element involved in defense and stress responsiveness; TATC-box:cis-acting element involved in gibberellin-responsiveness; ACE:cis-acting element involved in light responsiveness; LTR:cis-acting element involved in low-temperature responsiveness; TCA-element:cis-acting element involved in salicylic acid responsiveness; ABRE:cis-acting element involved in the abscisic acid responsiveness; A-box:cis-acting regulatory element; ARE:cis-acting regulatory element essential for the anaerobic induction; AuxRR-core:cis-acting regulatory element involved in auxin responsiveness; circadian:cis-acting regulatory element involved in circadian control; G-box:cis-acting regulatory element involved in light responsiveness; RY-element:cis-acting regulatory element involved in seed-specific regulation; CGTCA-motif:cis-acting regulatory element involved in the MeJA-responsiveness; TGACG-motif:cis-acting regulatory element involved in the MeJA-responsiveness;O2-site:cis-acting regulatory element involved in zein metabolism regulation; CAT-box:cis-acting regulatory element related to meristem expression; GCN4_motif:cis-regulatory element involved in endosperm expression; GARE-motif:gibberellin-responsive element; P-box:gibberellin-responsive element; 3-AF1

binding site:light responsive element; GT1-motif:light responsive element; Sp1:light responsive element; MBS:MYB binding site involved in drought-inducibility; MBSI:MYB binding site involved in flavonoid biosynthetic genes regulation; MRE:MYB binding site involved in light responsiveness; CCAAT-box:MYBHv1 binding site; 3-AF3 binding site:part of a conserved DNA module array (CMA3); ATC-motif:part of a conserved DNA module involved in light responsiveness; ATCT-motif:part of a conserved DNA module involved in light responsiveness; Box 4:part of a conserved DNA module involved in light responsiveness; chs-CMA1a:part of a light responsive element; chs-CMA2a:part of a light responsive element; GA-motif:part of a light responsive element; GATA-motif:part of a light responsive element; I-box:part of a light responsive element; LAMP-element:part of a light responsive element; TCCC-motif:part of a light responsive element; TCT-motif:part of a light responsive element; AT1-motif:part of a light responsive module; AE-box:part of a module for light response; AuxRE:part of an auxin-responsive element; TGA-box:part of an auxin-responsive element; HD-Zip 3:protein binding site; A-box:sequence conserved in alpha-amylase promoters.

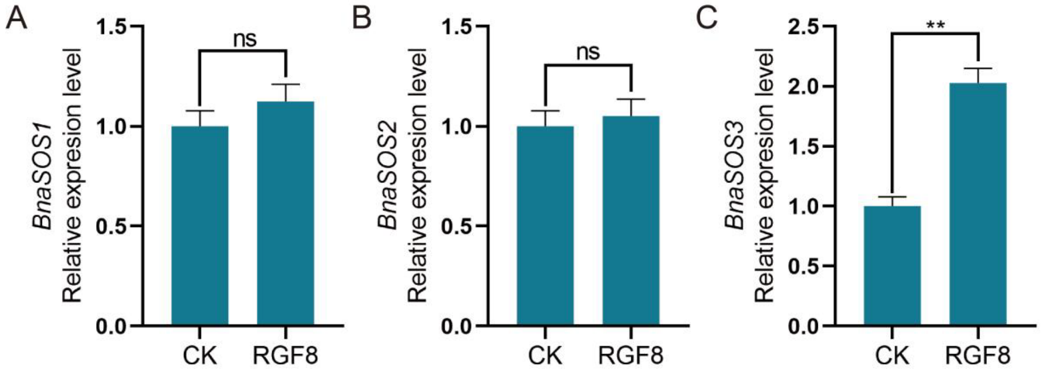

Figure S4. RT-qPCR analysis of BnaSOSs. **(A-C)** Differential Responses of *BnaSOSs*. Expression Under BnRGF8 treatment in *Brassica napus* Seedlings. The expression levels of each *BnaSOS* gene in the BnRGF8 treatment group were normalized to those in the control group (CK). Each bar data represents are mean ± standard error (n = 3). Statistical analysis was performed with Student's t-test (* $p < 0.05$; ** $p < 0.01$).

Table S1. Conserved motifs of BnRGF protein.

Motif	Coding sequence length (aa)	Amino acid sequence(5,-3,)
Motif 1	14	MDYPQPRRKPPIHN
Motif 2	44	LALLLIFFICSPAQARNIGGIVRKRTLMVVEKDQETRNSWQDGG
Motif 3	41	IAQNNEEITKLEVPSTNVTKTLLSEAPIEHAVGBHGEIIEK
Motif 4	40	MVFCFILLLLSSNVGCANARRLGFHKHHHKIASLVQSVVR
Motif 5	50	HPKDSHDFTNTGAPRKLRPGRTMRTTVVIAKKEKVRATNNDDLSIKISGG
Motif 6	29	SVSSKKVSSSWKVKKKSKKLPRSDEEHPK
Motif 7	50	HLMMERKVGFHKRSKSSSFKWKPNKKKASGRFVAFYDDYRGPASHPPRHN
Motif 8	21	MMRFTIJJLAFLIJJQALSDD
Motif 9	15	ILVYAHEGGDAGHKS
Motif 10	21	PKKPIEKETGVEEEDDLVAYT

Table S2. Cis-acting elements.

Cis-acting elements.	Sequence	Cis-acting elements.
TGA-element	AACGAC	auxin-responsive element
TGACG-motif	TGACG	cis-acting regulatory element involved in the MeJA-responsiveness
TGA-box	TGACGTAA	part of an auxin-responsive element
TCT-motif	TCTTAC	part of a light responsive element
TC-rich repeats	GTTTTCTTAC	cis-acting element involved in defense and stress responsiveness
TCCC-motif	TCTCCCT	part of a light responsive element
TCA-element	CCATCTTTTT	cis-acting element involved in salicylic acid responsiveness
TATC-box	TATCCCA	cis-acting element involved in gibberellin-responsiveness
Sp1	GGGCGG	light responsive element
RY-element	CATGCATG	cis-acting regulatory element involved in seed-specific regulation
P-box	CCTTTTG	gibberellin-responsive element
O2-site	GATGACATGG	cis-acting regulatory element involved in zein metabolism regulation
MSA-like	TCAAACGGT	cis-acting element involved in cell cycle regulation
MRE	AACCTAA	MYB binding site involved in light responsiveness
MBSI	aaaAaaC(G/C)GTTA	MYB binding site involved in flavonoid biosynthetic genes regulation
MBS	CAACTG	MYB binding site involved in drought-inducibility
LTR	CCGAAA	cis-acting element involved in low-temperature responsiveness
LAMP-element	CTTTATCA	part of a light responsive element
I-box	CATATCCAAT	part of a light responsive element
HD-Zip 3	GTAAT(G/C)ATTAC	protein binding site
GT1-motif	GGTTAA	light responsive element
GT1-motif	GGTTAAT	light responsive element
GCN4_motif	TGAGTCA	cis-regulatory element involved in endosperm expression
G-box	CACGTG	cis-acting regulatory element involved in light responsiveness
G-box	TACGTG	cis-acting regulatory element involved in light responsiveness
G-box	TAAACGTG	cis-acting regulatory element involved in light responsiveness
G-box	TCCACATGGCA	cis-acting regulatory element involved in light responsiveness
GATA-motif	AAGATAAGATT	part of a light responsive element

GARE-motif	TCTGTTG	gibberellin-responsive element
GA-motif	ATAGATAA	part of a light responsive element
circadian	CAAAGATATC	cis-acting regulatory element involved in circadian control
chs-CMA2a	TCACTTGA	part of a light responsive element
chs-CMA1a	TTACTTAA	part of a light responsive element
CGTCA-motif	CGTCA	cis-acting regulatory element involved in the MeJA-responsiveness
CCAAT-box	CAACGG	MYBHv1 binding site
CAT-box	GCCACT	cis-acting regulatory element related to meristem expression
Box 4	ATTAAT	part of a conserved DNA module involved in light responsiveness
AuxRR-core	GGTCCAT	cis-acting regulatory element involved in auxin responsiveness
AuxRE	TGTCTCAATAAG	part of an auxin-responsive element
AT-rich element	ATAGAAATCAA	binding site of AT-rich DNA binding protein (ATBP-1)
ATCT-motif	AATCTAATCC	part of a conserved DNA module involved in light responsiveness
ATC-motif	AGTAATCT	part of a conserved DNA module involved in light responsiveness
AT1-motif	AATTATTTTTTATT	part of a light responsive module
ARE	AAACCA	cis-acting regulatory element essential for the anaerobic induction
AE-box	AGAAACAA	part of a module for light response
ACE	CTAACGTATT	cis-acting element involved in light responsiveness
ABRE	GACACGTGGC	cis-acting element involved in the abscisic acid responsiveness
A-box	CCGTCC	cis-acting regulatory element
A-box	AATAACAAACTCC	sequence conserved in alpha-amylase promoters
3-AF3 binding site	CACTATCTAAC	part of a conserved DNA module array (CMA3)
3-AF1 binding site	TAAGAGAGGAA	light responsive element

Table S3. *B. napus* used in this study

Material ID	Features	flowering time (days)	Plant height (m)
Y110	yellow flower	135	1.2
Y124	yellow flower	155	1.2
Y154	yellow flower, good taste	140	1.25
Y167	yellow flower	123	1.26
Y343	purple leaves,	168	2.25
Y366	lobed leaves with white flowers	156	1.4
Y56	more branches	155	1.2
Y94	yellow flower	145	1.53
Y121	good plant shape	150	1.26
Y100	yellow flowers with red center	148	1.12

Table S4. Primers used in this study

**Primers name**	Primer sequence（5'-3'）
BnaRGF2-F	TCTTTTGTTTTGCCTCTCGTG
BnaRGF2-R	TCTATTGGCTTCTTCGGATTT
BnaRGF3-F	TGTGCGTTGATCATTCTTTTCTTA
BnaRGF3-R	TTTCTTTATCTGCTTCTCCATTTG
BnaRGF4-F	TTTCCTTATTATCATTCATGCCCT
BnaRGF4-R	ATTATTCGTCGCCCTTACTTTCTC
BnaRGF5-F	CAAGGATCAAAATGTTGTCCCC
BnaRGF5-R	GTAATCTGCAAATAAGAGCGGC
BnaRGF6-F	AAAGGAGGGTGCTTGGTGGA
BnaRGF6-R	GCTTACGATGAGGCTGAGGG
BnaRGF8-F	ACTCCACCCTCGCTACTCATC
BnaRGF8-R	GCATTCTCCTCTTCCTTTTCA
BnaRGF9-F	TCATCTTCTTCATTTGTTCTCCTGC
BnaRGF9-R	ATTTGCGATTATGTATCGGTCTTTT
BnaACT7-F	CTTCCTCACGCTATCCTCCG
BnaACT7-R	AGCCGTCTCCAGCTCTTGC
BnaRGF11-F	ATGCATCAAAACCCTAATCCTCC
BnaRGF11-R	CGGTGCTTCGCTTGATATGGTTT
BnaRGF13-F	AGTTGTGAAGTTACAGACACTCCT
BnaRGF13-R	CTGTTGGCACTGAATCTTTCAACG
BnaCYCB1;1-F	TCTTCCTCGCACGTTTCATCA
BnaCYCB1;1-R	ACGCCAATAGCTTCGCACAAT
BnaSOS1-F	TAATCAAGGAGAGTGAAACCGAA
BnaSOS1-R	GACAGCATCATGAAGATGAGCGA
BnaSOS2-F	ATTAGATCTTTCCGGTTTGTTTG
BnaSOS2-R	TATATTCCGATTGTGAATTTCC
BnaSOS3-F	CGGTCTTTAGGTGTCTTCCATCC
BnaSOS3-R	CATTTCTTTCAATTCTTCTCGCT
